# Pharmacokinetics of isoflavones, daidzein and genistein, after ingestion of soy beverage compared with soy extract capsules in postmenopausal Thai women

**DOI:** 10.1186/1472-6904-5-2

**Published:** 2005-03-03

**Authors:** Ekasin Anupongsanugool, Supanimit Teekachunhatean, Noppamas Rojanasthien, Saipin Pongsatha, Chaichan Sangdee

**Affiliations:** 1 Department of Pharmacology, Faculty of Medicine, Chiang Mai University, Thailand; 2 Department of Obstetrics and Gynecology, Faculty of Medicine, Chiang Mai University, Thailand

## Abstract

**Background:**

Isoflavones from soybeans may provide some beneficial impacts on postmenopausal health. The purpose of this study was to compare the pharmacokinetics and bioavailability of plasma isoflavones (daidzein and genistein) after a single dose of orally administered soy beverage and soy extract capsules in postmenopausal Thai women.

**Methods:**

We conducted a randomized two-phase crossover pharmacokinetic study in 12 postmenopausal Thai women. In the first phase, each subject randomly received either 2 soy extract capsules (containing daidzin : genistin = 7.79 : 22.57 mg), or soy beverage prepared from 15 g of soy flour (containing daidzin : genistin = 9.27 : 10.51 mg). In the second phase, the subjects received an alternative preparation in the same manner after a washout period of at least 1 week. Blood samples were collected immediately before and at 0.5, 1, 2, 4, 6, 8, 10, 12, 24 and 32 h after administration of the soy preparation in each phase. Plasma daidzein and genistein concentrations were determined by using high performance liquid chromatography (HPLC). The pharmacokinetic parameters of daidzein and genistein, i.e. maximal plasma concentration (C_max_), time to maximal plasma concentration (T_max_), area under the plasma concentration-time curve (AUC) and half-life (t_1/2_), were estimated using the TopFit version 2.0 software with noncompartmental model analysis.

**Results:**

There were no significant differences in the mean values of C_max_/dose, AUC_0–32_/dose, AUC_0-∝_/dose, T_max_, and t_1/2 _of genistein between both preparations. For pharmacokinetic parameters of daidzein, the mean values of C_max_/dose, T_max_, and t_1/2 _did not significantly differ between both preparations. Nonetheless, the mean AUC_0–32_/dose and AUC_0-∝_/dose after administration of soy extract capsules were slightly (but significantly, p < 0.05) higher than those of soy beverage.

**Conclusion:**

The bioavailability of daidzein, which was adjusted for the administered dose (AUC/dose), following a single oral administration of soy beverage was slightly (but significantly) less than that of soy extract capsules, whereas, the bioavailability adjusted for administered dose of genistein from both soy preparations were comparable. The other pharmacokinetic parameters of daidzein and genistein, including C_max _adjusted for the dose, T_max _and t_1/2_, were not different between both soy preparations.

## Background

Menopause is associated with estrogen deficiency and its accompanying symptoms such as accelerated bone loss and atherosclerosis. Hormone replacement therapy (HRT) has traditionally been used for treatment of menopausal disorders. Estrogen helps to maintain bone density, relieve menopausal symptoms, as well as influence emotional state [1]. Estrogen replacement therapy after menopause therefore improves the health and quality of life for women. However, the Women's Health Initiative (WHI) randomized controlled trial has recently found that although estrogen-alone hormone therapy reduces the risk of hip and other fractures in healthy postmenopausal women with prior hysterectomy, it significantly increases the risk of stroke (but has no significant effect on the risk of coronary heart disease, breast or colorectal cancer) [2]. In addition, long-term estrogen replacement therapy in postmenopausal women who have a uterus might has the disadvantage of being tissue agonists for endometrial tissue, which increases the incidence of endometrial cancer [3]. Although adding progestin to estrogen can be used to prevent the development of endometrial cancer, this combination may cause some unwanted side effects, i.e. breast cancer, venous thromboembolism, stroke and coronary heart disease [3]. Thus, alternative therapies, which include natural products such as phytoestrogens and herbs, offer attractive options because they may protect against breast and endometrial cancer, have fewer side effects and still provide health benefits [4].

**Figure 1:**
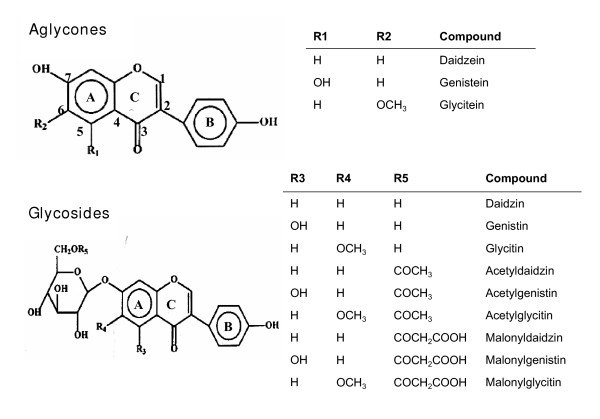
Structures of isoflavones.

The most common forms of phytoestrogens are the isoflavones. Among the food consumed by humans, soybeans contain the highest concentration of isoflavones. Isoflavones have a chemical structure resembling that of estrodiol-17β, the most potent mammalian estrogen. The major isoflavones, namely, genistein and daidzein, have several features in common with estradiol-17β, including an aromatic A ring with hydroxyl group in the same plane at a distance similar to that in estradiol (Figure 1) [5]. Indeed, isoflavones appear to have both estrogenic and antiestrogenic effects, like selective estrogen receptor modulators (SERMs), depending on the target tissue [6]. Therefore, rather than classifying soy isoflavones as "estrogens", they should be judged more correctly as natural SERMs. Reproductive cells, especially those of the breast and uterus, are rich in estrogen receptor α (ERα), whereas other cells (such as those in the bone) have greater amounts of estrogen receptor β (ERβ) than ERα. This differential distribution of the two types of estrogen receptors, and the greater affinity of the isoflavones for ERβ in relation to ERα, suggests that the isoflavones have different effects in different tissue [7].

Despite the beneficial effects of isoflavones on postmenopausal health are still controversial, there are some researches, including epidemiological studies, suggest that isoflavones may help to alleviate postmenopausal symptoms and protect against chronic diseases such as hormone-dependent cancer (e.g. breast and endometrial cancer), cardiovascular diseases and osteoporosis [8-11]. Thus, in terms of both health promotion and chronic disease prevention, the potential public health impact of daily soy consumption could be important, especially in postmenopausal women.

Although many commercial soy capsules containing isoflavone extract are now available in many Western countries, soybeans and soy food (such as tofu, soy flour, soy milk, etc.), which provide the main sources of isoflavones, are consumed in significant amounts in Asian countries because they are inexpensive and high in quality protein. The purpose of this trial was to compare the pharmacokinetics of plasma isoflavones, daidzein and genistein, in postmenopausal Thai women after a single dose of orally administered commercial soy extract capsules and soy beverage.

## Methods

### Study design

This study was a single dose, randomized two-phase crossover study with a washout period of at least one week. It was approved by the Medical Ethics Committee of the Faculty of Medicine, Chiang Mai University and was in compliance with the Helsinki Declaration.

### Subjects

A total of 12 postmenopausal Thai women, who ranged in age from 46–61 years (average 52.83 ± 3.88 years), participated in this study. Their mean weight and height was 52.23 ± 6.38 kg and 1.54 ± 0.06 cm, respectively. The body mass index (BMI) of each subject was within 18–24 kg/m^2 ^(22.06 ± 1.83 kg/m^2^). Their serum follicle-stimulating hormone concentrations were more than 20 IU/l and the average level was 69.60 ± 31.21 IU/l. All had to be in good health on the basis of medical history and physical examination. Routine blood tests including complete blood count (CBC) with differential, blood urea nitrogen (BUN), creatinine (Cr) and liver function test (LFT) had to be within the normal limit. Subjects had to give both verbal and written information regarding the study. Signed informed consent was obtained prior to entry. Exclusion criteria included subjects with known premenopausal status (<12 months since the last spontaneous menstrual bleeding and a serum follicle-stimulating hormone concentration ≤ 20 IU/l) as well as those with a known history of chronic renal, liver, pulmonary or cardiovascular diseases, recent cigarette smoking, substance abuse or addiction, use of antibiotics within the previous 6 weeks, consumption of more than 2 alcoholic drinks/day, regular use (more than 1 dose/week) of over-the-counter or prescribed medications, and malignancy.

### Isoflavone preparations

The isoflavone preparations used in this study were commercial soy extract capsules and soy beverage. Soy extract capsules were purchased from Canada, whereas, the soy beverage was prepared from mixing 15 g of commercial soy flour (Doi Khum^®^, Chiang Mai, Thailand, lot no. 19GODE) with 300 ml of hot water. The concentration of isoflavones in each preparation was measured as aglycones (daidzein and genistein) and β-glycoside conjugates of isoflavones (daidzin and genistin) that were analyzed by high performance liquid chromatography (HPLC) as described below.

### Quantification of isoflavones in soy preparations

Two soy preparations, soy flour and soy extract capsules, were chosen as isoflavone sources. The sample extractions and concentration determinations were modified from the method described by Nakamura et al. [12]. Two hundred mg of soy flour or 300 mg of powder from the soy capsule (1 capsule) was placed in a centrifuge tube. Ten ml of 80% methanol in water was added to the centrifuged tubes, and sonicated for 30 min. Isoflavonoids were extracted for 24 h at an ambient temperature. One ml of the mixture was centrifuged, and 10 μl of clear supernatant was diluted with mobile phase (100 times for soy flour and 400 times for soy extract capsule) and spiked with 20 μl of internal standard (IS, 100,000 ng/ml fluorescein and 50,000 ng/ml chloramphenicol for quantification of aglycones and β-glycosides, respectively). Five μl of the mixture was injected into the HPLC system. Separation was performed isocratically at 50°C. The flow rate of the mobile phase was maintained at 1 ml/min and the analytes were detected by UV absorption at 259 nm. The mobile phase for the quantification of aglycones consisted of 5 mM phosphoric acid in methanol/acetonitrile (100:85, v/v), whereas, that for quantification of β-glycoside conjugates comprised 5 mM phosphoric acid in methanol/acetonitrile (80:20, v/v). The Isoflavone contents of unknown samples were determined by using a calibration curve of the peak height ratios of isoflavones and IS versus respective isoflavone concentrations with the use of linear regression. All samples were analyzed within the same day, in which intraday assay validation was performed.

### Dosage and drug administration

Subjects were admitted to the Clinical Pharmacology Unit of the Faculty of Medicine, Chiang Mai University at 6:30 a.m. after an overnight fast of at least 8 h. They were randomized to receive either 2 soy extract capsules with 300 ml of water, or 300 ml of soy beverage at 7:00 a.m. They remained upright and fasted for 2 h after soy product administration. Water and lunch were served at 2 h and 4 h, respectively after dosing. Blood samples were collected at different time points (see below). After the blood sample collection at 12 h postdose, the subjects were discharged from the Clinical Pharmacology Unit and asked to come back again on the next day to give blood samples at 24 and 32 h postdose. While waiting for blood sample collections, the subjects were allowed to perform all of their daily activities, except moderate to high degrees of exercises. After a washout period of at least 1 week, the subjects received the alternative preparation and the blood samples were collected in the same manner. Identical food and fluid were served during the 2 study periods. The subjects were required to refrain from drinking caffeine containing beverages and alcohol, and instructed to consume no soy products (except those given in this study) from the time of screening until the end of the research.

### Blood sample collection

Venous blood samples (7 ml/each) for determination of soy isoflavones were collected predose and then exactly 0.5, 1, 2, 4, 6, 8, 10, 12, 24 and 32 h after administration. Samples were obtained from the forearm by venipuncture through an indwelling intravenous catheter (BD Insyte^®^) and collected in a heparinized vacutainer (BD Insyte^®^). The blood collecting tubes were centrifuged at 2,500 rpm for 20 min and the plasma samples were separated and frozen at -20°C until analyzed.

### Determination of plasma isoflavone concentrations

The assay was modified from the solid phase extraction procedure and HPLC technique as previously described by Thomas et al [13]. Briefly, an aliquot (125 μl) of plasma was transferred to a 1.5 ml plastic vial and treated with 0.25 ml of β-glucuronidase/sulfatase mixture from *Helix pomatia *(Sigma G-0876) to hydrolyze glucuronide and sulfate conjugates of genistein and daidzein. The enzyme mixture was made up freshly and contained 0.15 g of ascorbic acid in 10 ml of 0.2 M acetate buffer, pH 4.0, and 500 μl of β-glucuronidase/sulfatase from *Helix pomatia*. To allow for complete hydrolysis in the plasma samples, 0.75 ml of 0.2 M ammonium acetate buffer was added, and the tubes were capped and heated overnight in a water bath (15–18 h, 37°C). The tubes were removed from the water bath and allowed to cool to room temperature. After enzymatic hydrolysis, plasma samples were spiked with 5 μl of internal standard (IS, 100,000 ng/ml fluorescein in 80% methanol). After vortex mixing for 30 sec and centrifugation at 13,000 rpm for 5 min, sample purification was performed by using a solid phase extraction cartridge placed in the vacuum box. The cartridges were preconditioned with 2.50 ml each of methanol, water and 175 mM phosphate buffer, respectively. The samples were loaded into the cartridges and allowed to flow freely, then the cartridges were washed with 0.1 ml of 20% methanol in water, 0.1 ml of 175 mM phosphate buffer and 0.1 ml of 20% methanol in water, consecutively. After drying the cartridge by slow suction, isoflavones and IS were eluted with 2.0 ml of 20% methanol in ethyl acetate. The eluents were dried by a SpeedVac concentrator. The residues were dissolved in 50 μl of the mobile phase, and 10 μl of samples were injected into a HPLC system. The mobile phase consisted of 4 mM perchloric acid in methanol/acetonitrile (115:85, v/v). The flow rate was maintained at 1 ml/min and the analytes were detected by UV absorption at 259 nm, and the column was maintained at 40°C. The retention time for daidzein and genistein was 7.699 and 13.031 minutes, respectively. The lower limit of quantitation was 5 ng/ml. Plasma concentrations of daidzein and genistein were determined using a calibration curve and linear regression of the seven known isoflavone concentrations versus the peak height ratios of isoflavones and IS.

The %CV of intraday precision for plasma daidzein concentrations ranged from 0.85–9.45%, whereas, the %CV of interday ranged from 3.90–9.96%. The %CV of intraday and interday precision for plasma genistein concentrations ranged from 1.29–3.65%, and 4.37–6.84%, respectively. The %deviation in intraday and interday assay for plasma daidzein concentrations ranged from 4.35–5.84% and -6.11–4.22%, respectively. On the other hand, the %deviation in intraday and interday assay for plasma genistein concentrations ranged from -5.00–1.98% and -7.79%–1.52%, respectively.

### Data analysis and statistical methods

#### Pharmacokinetic parameters

The maximal plasma concentration (C_max_, ng/ml) and time to maximal plasma concentration (T_max_, h) were obtained directly by the visual inspection of each subject's plasma concentration-time profile. The areas under the plasma concentration-time curve from time 0–32 (AUC_0–32_, ng.h/ml) and 0-∝ (AUC_0-∞_, ng.h/ml) as well as half-life (t_1/2_, h) were determined by non-compartmental analysis. The slope of the terminal log-linear portion of the concentration-time profile was determined by least-squares regression analysis and used as the elimination rate constant (*K*_*e*_). The elimination t_1/2 _was calculated as 0.693/*K*_*e*_. The AUC from time zero to the last quantifiable point (AUC_0–32_) was calculated using the trapezoidal rule. Extrapolated AUC from time t to infinity (AUC_t-∞_) was determined as Ct/*K*_*e*_. Total AUC was the sum of AUC_0–32+ _AUC_32-∞_. In this study, the sampling time was continued for more than 3 half-lives, therefore, the AUC_0-32 _was sufficient to cover at least 80% of the total AUC. The calculation was performed by using the TopFit software version 2.0 for personal computer.

#### Statistical analysis

The pharmacokinetic parameters were presented as mean ± SD. The differences between the mean values of C_max_/dose, T_max_, t_1/2_, AUC_0–32_/dose and AUC_0-∝_/dose of the two isoflavone preparations were statistically analyzed by using the paired *t*-test. However, the results from the analysis were not different regardless of whether the statistical comparison was performed by using the paired *t*-test (parametric method) or Wilcoxon's sign rank test (non-parametric method).

## Results

### Isoflavone contents in soy preparations

The quantification of isoflavone contents demonstrated that both soy flour and soy extract capsule preparations contained predominantly daidzin and genistin (the form of β-glycoside conjugates), whereas, aglycones were rarely found and isoflavones in other forms were not measured. Isoflavone contents in both soy preparations are shown in Table 1.

**Table 1:** Isoflavone contents in soy preparations used in this study.

	Daidzin	Genistin	Total^1^
Soy flour			
Mg/g	0.62 ± 0.005	0.70 ± 0.01	1.32 ± 0.01
%	47	53	100
Soy extract capsule			
Mg/capsule	3.90 ± 0.04	11.29 ± 0.17	15.18 ± 0.21
%	26	74	100

^1^Summation of daidzin and genistin contents.

### Pharmacokinetics of daidzein and genistein in healthy postmenopausal Thai women

**Figure 2:**
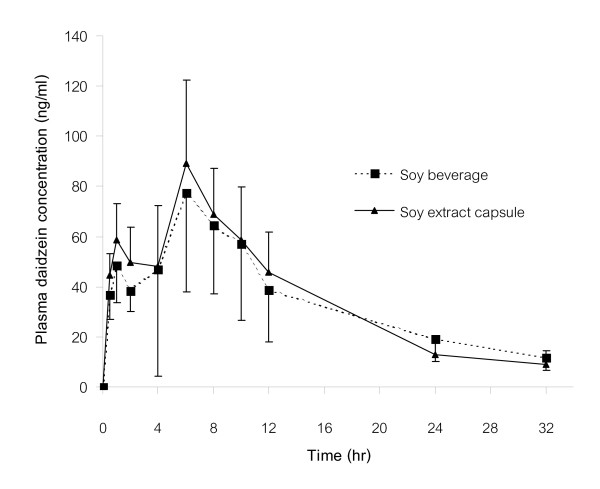
Mean plasma daidzein concentration-time curves after a single dose of orally administered soy beverage and soy extract capsules.

The Mean plasma daidzein and genistein concentration-time curves after a single dose of both orally administered soy preparations are shown in Figure 2, 3. The individual and mean pharmacokinetic parameters of daidzein and genistein following oral administration of soy beverage and soy extract capsules are shown in Tables 2, 3.

**Figure 3:**
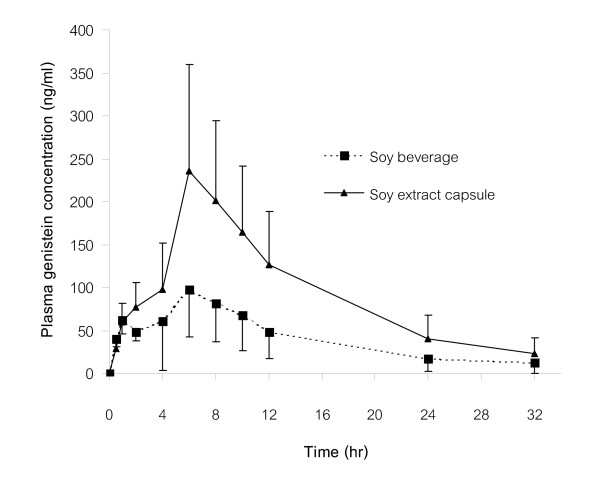
Mean plasma genistein concentration-time curves after a single dose of orally administered soy beverage and soy extract capsules. (Note: the orally administered dose of genistein from soy extract capsules was approximately two times higher than that from soy beverage).

**Table 2:** Individual and mean pharmacokinetic parameters of daidzein following a single dose of orally administered soy beverage (B) and soy extract capsules (C).

Subj No.	C_max _(ng/ml)		C_max_/Dose^1^		AUC_0–32 _(ng.h/ml)		AUC_0–32_/Dose^1^		AUC_0-∝ _(ng.h/ml)		AUC_0-∝_/Dose^1^		T_max _(h)		t_1/2 _(h)	
	
	B	C	B	C	B	C	B	C	B	C	B	C	B	C	B	C
1	67.42	60.73	7.27	7.80	354.62	650.40	38.25	83.49	496.21	722.51	53.53	92.75	6	6	6.06	8.04
2	142.17	56.33	15.34	7.23	1142.38	931.84	123.23	119.62	1206.09	1024.24	130.11	131.48	6	1	6.74	6.02
3	64.95	76.70	7.01	9.85	1118.40	1117.20	120.65	143.41	1704.60	1171.78	183.88	150.42	8	6	19.80	6.85
4	132.30	124.43	14.27	15.97	1336.29	1356.49	144.15	174.13	1402.26	1427.11	151.27	183.20	6	6	6.70	6.91
5	77.66	93.20	8.38	11.96	1293.52	1251.35	139.54	160.64	1483.69	1561.72	160.05	200.48	8	10	9.94	10.50
6	105.65	142.82	11.40	18.33	1409.24	1378.43	152.02	176.95	1533.26	1450.55	165.40	186.21	10	6	6.21	6.86
7	160.86	125.37	17.35	16.09	1576.65	1669.28	170.08	214.28	1761.36	1721.18	190.01	220.95	4	6	8.48	5.97
8	102.77	74.08	11.09	9.51	1158.07	1090.36	124.93	139.97	1227.07	1149.04	132.37	147.50	6	6	6.85	6.76
9	45.34	105.77	4.89	13.58	300.89	849.39	32.46	109.04	340.35	876.40	36.72	112.50	4	6	3.46	4.40
10	52.98	84.74	5.72	10.88	401.82	579.95	43.35	74.45	533.03	761.42	57.50	97.74	1	6	4.82	4.38
11	99.98	87.38	10.79	11.22	1291.80	1546.66	139.35	198.54	1455.80	1705.46	157.04	218.93	8	10	6.59	7.63
12	103.60	120.64	11.18	15.49	578.60	909.87	62.42	116.80	665.06	971.69	71.74	124.74	4	6	6.49	5.71

**Mean**	96.31	96.02^ND^	10.39	12.33	996.86	1110.94^ND^	107.54	142.61*	1150.73	1211.93^ND^	124.14	155.57*	5.92	6.25	7.68	6.67

**SD**	36.18	27.71	3.90	3.56	455.69	342.26	49.16	43.94	504.94	354.46	54.47	45.50	2.43	2.26	4.14	1.65

^1^Pharmacokinetic parameters adjusted for oral administered dose. ND = statistical analysis was not performed due to differences in oral administered doses between both preparations, *Statistical significance (p < 0.05) compared to corresponding values of soy extract capsules.

**Table 3:** Individual and mean pharmacokinetic parameters of genistein following a single dose of orally administered soy beverage (B) and soy extract capsules (C).

Subj No.	C_max _(ng/ml)		C_max_/Dose^1^		AUC_0–32 _(ng.h/ml)		AUC_0–32_/Dose^1^		AUC_0-∝ _(ng.h/ml)		AUC_0-∝_/Dose^1^		T_max _(h)		t_1/2 _(h)	
	
	B	C	B	C	B	C	B	C	B	C	B	C	B	C	B	C
1	90.41	151.49	8.60	6.71	954.82	1950.40	90.85	86.42	1040.69	2248.09	99.02	99.61	6	6	8.46	9.92
2	68.08	124.90	6.48	5.53	693.31	1821.17	65.97	80.69	739.89	1941.50	70.40	86.02	1	8	7.62	7.42
3	149.53	301.34	14.23	13.35	1801.34	4392.34	171.39	194.61	1989.66	5048.92	189.31	223.70	8	8	8.25	9.37
4	212.94	516.77	20.26	22.90	1979.53	5541.88	188.35	245.54	2140.25	6175.55	203.64	273.62	6	6	8.10	8.93
5	96.95	220.38	9.22	9.76	1281.58	2478.42	121.94	109.81	1423.11	3072.33	135.41	136.12	8	10	9.73	12.40
6	96.57	301.62	9.19	13.36	1277.75	2776.28	121.57	123.01	1483.87	2972.50	141.19	131.70	10	6	10.10	7.74
7	223.77	371.31	21.29	16.45	3184.25	5260.83	302.97	233.09	4022.02	6003.94	382.69	266.01	4	8	12.60	9.58
8	110.60	193.01	10.52	8.55	948.75	2217.44	90.27	98.25	998.31	2370.24	94.99	105.02	6	6	5.07	8.41
9	54.86	183.88	5.22	8.15	425.80	1481.47	40.51	65.64	527.69	1500.60	50.21	66.49	4	6	4.41	4.80
10	69.95	214.54	6.66	9.51	773.90	1856.73	73.63	82.27	789.07	1940.05	75.08	85.96	6	6	5.34	6.63
11	105.13	217.57	10.00	9.64	1128.81	3121.71	107.40	138.31	1177.74	3208.73	112.06	142.17	6	8	6.32	5.27
12	117.61	345.31	11.19	15.30	871.60	2591.36	82.93	114.81	914.46	2631.99	87.01	116.61	4	6	5.35	5.05

**Mean**	116.37	261.84^ND^	11.07	11.60	1276.79	2957.50^ND^	121.48	131.04	1437.23	3259.54^ND^	136.75	144.42	5.75	7.00	7.61	7.96

**SD**	53.80	110.68	5.12	4.90	746.04	1372.13	70.98	60.79	949.02	1599.34	90.30	70.86	2.34	1.35	2.44	2.28

^1^Pharmacokinetic parameters adjusted for oral administered dose. ND = statistical analysis was not performed due to differences in oral administered doses between both preparations.

The mean plasma daidzein concentration-time curve after a single dose of both orally administered soy preparations revealed a biphasic pattern. The first peak of plasma daidzein concentration was reached approximately 1 h after ingestion of both preparations, whereas, the second peak attained higher plasma concentrations at 5.92 ± 2.43 h for soy beverage, and 6.25 ± 2.26 h for soy extract capsules. The mean maximal plasma daidzein concentrations (C_max_) were 96.31 ± 36.18 ng/ml and 96.02 ± 27.71 ng/ml for soy beverage and soy extract capsules, respectively. Pharmacokinetic analysis of the plasma concentration-time curves showed that the elimination t_1/2 _was 7.68 ± 4.14 h for soy beverage and 6.67 ± 1.65 h for soy extract capsules. The AUC_0–32 _was 996.86 ± 455.69 and 1110.94 ± 342.26 ng.h/ml for soy beverage and soy extract capsules, respectively, whereas, the AUC_0-∝ _was 1,150.73 ± 504.94 ng.h/ml for soy beverage and 1,211 ± 354.46 ng.h/ml for soy extract capsules.

The mean plasma genistein concentration-time curve after a single dose of both orally administered soy preparations also demonstrated a biphasic pattern, but the first peak of plasma genistein concentration after ingestion of soy extract capsules was less evident. The mean T_max _for the second peak of plasma concentration was 5.75 ± 2.34 h for soy beverage and 7.00 ± 1.35 h for soy extract capsules. The mean C_max _was 116.37 ± 53.80 ng/ml for soy beverage and 261.84 ± 110.68 ng/ml for soy extract capsules. Pharmacokinetic analysis of the plasma concentration-time curves showed the elimination t_1/2 _of 7.61 ± 2.44 h for soy beverage and 7.96 ± 2.28 h for soy extract capsules. The AUC_0–32 _was 1276.79 ± 746.04 and 2957.50 ± 1372.13 ng.h/ml for soy beverage and soy extract capsules, respectively, whereas, the AUC_0-∝ _was 1,437.23 ± 949.02 ng.h/ml for soy beverage and 3,259.54 ± 1,599.34 ng.h/ml for soy extract capsules.

After oral administration of both soy preparations, the pharmacokinetic parameters of daidzein were statistically compared, and the mean values of C_max_/dose, T_max_, and t_1/2 _did not significantly differ between both preparations. Nonetheless, the mean values of AUC_0–32_/dose and AUC_0-∝_/dose after administration of soy extract capsules were slightly (but significantly, p < 0.05) higher than those after soy beverage intake (Table 2). For pharmacokinetic parameters of genistein, there were no significant differences in the mean values of C_max_/dose, AUC_0–32_/dose, AUC_0-∝_/dose, T_max_, and t_1/2 _between both preparations (Table 3).

## Discussion

In this study, the pharmacokinetics of plasma daidzein and genistein were evaluated in 12 postmenopausal Thai women after a single dose of orally administered soy beverage and soy extract capsules. These women were enrolled from a pool of volunteers after they had been screened for medical history, BMI, serum follicle-stimulating hormone concentration and blood tests. Since the design of this study was similar to that of the bioequivalence testing, 12 subjects were enrolled according to the minimum number of subjects stipulated by the Canadian and European guidelines for bioequivalence testing.

The soy extract product available in Thailand was not selected as the study preparation because only minimum isoflavone content was labeled without any details about the proportion of daidzin and genistin content. The Canadian soy extract preparation was used as an alternative because the exact total of isoflavone content (18.2 mg/capsule) as well as daidzin and genistin content (9.1 mg/capsule, each) were declared. Nonetheless, our quantification of isoflavones in this preparation revealed that the amount of daidzin and genistin in each capsule was 3.90 ± 0.04 and 11.29 ± 0.17, respectively. Therefore, the orally administered dose in this study was calculated according to our quantification, but not by the amount declared.

Initially, we tried to measure the total isoflavone contents in each preparation by using the acid hydrolysis method [14,15]. Briefly, either 1 g of soy flour or powder from the soy extract capsules was dispersed in a mixture of 10 ml of 10 M HCl and 40 ml of 96% ethanol (containing 0.05% butylated hydroxy toluene, BHT) followed by refluxing at 100°C for 2 h. The mixture was cooled to room temperature and the ethanol lost during the refluxing was replaced. One ml of this mixture was centrifuged. Ten μl of clear supernatant was diluted 100 times and determined by the HPLC method. The principle of this assay is to hydrolyze β-glycoside conjugates of isoflavones to aglycones, and the detection of total aglycones reflects the total isoflavones. Unfortunately, we failed to measure total isoflavones as aglycones after acid hydrolysis. The recovery of daidzein and genistein was very low (approximately 20–30%) as compared to the other published data, even though we had tried to vary many factors such as the concentration of HCl, duration of refluxing time, temperature during reflux, amount of BHT added, etc. However, since isoflavones are present predominantly as β-glycoside conjugates (e.g. daidzin and genistin) in most commercially available soy products such as soybean or soy flour (with the exception of fermented soy products) [14], we used a specific HPLC condition for measuring daidzin and genistin, and another condition for measuring aglycones (daidzein and genistein) without acid hydrolysis. Isoflavones in other forms (malonyl glycoside and acetyl glycoside conjugates) were not determined because their commercial standards were not available. Glycitein and its derivatives were also not determined, due to a much smaller amount found in soybeans [16].

Since both soy preparations consist of different proportions of daidzin : genistin (approximately 1:1 for soy flour, 1:3 for soy extract capsules), the appropriate amount of daidzin content in each preparation was therefore considered first for pharmacokinetic comparison. In this study, each volunteer was assigned to receive 2 capsules of soy extract (daidzin : genistin = 7.79 : 22.57 mg) to compare with soy beverage prepared from 15 g of soy flour (9.27 : 10.51 mg). These dosages caused an approximately equal amount of daidzin between the two preparations, whereas, the genistin content in soy extract capsules was approximately two-fold higher than that of soy beverage. The oral administration of these dosages resulted in the plasma concentrations of daidzein and genistein being high enough and convenient for measurement by the HPLC method. In addition, preparing soy beverage from 15 g of soy flour in 300 ml of water was practical and created an acceptable concentration for consumption.

From previous studies, the bioavailability of isoflavones was investigated and compared among various soy food and beverages. So far, there has been no study that compares bioavailability of isoflavones from soy extract versus natural soy food or beverages. Our purpose was to investigate pharmacokinetics of daidzein and genistein after ingestion of soy beverage compared to soy extract capsules in postmenopausal Thai women. Daidzein and genistein contents in soy food can vary and depend on the raw material and processing conditions used to produce a particular food product. Furthermore, in each type of soy food, there are different forms of isoflavones in differing amounts. However, based on the equivalent dose of isoflavones, the administration of different soy food has shown no difference in isoflavone bioavailability [17]. In our study, the bioavailability, adjusted for dosage (determined by AUC_0–32_/dose and AUC_0-∝_/dose) of genistein after ingestion of both soy preparations, was not significantly different. In contrast, the bioavailability of daidzein following ingestion of soy extract capsules was significantly greater than that following ingestion of soy beverage. This might be the result of at least 2 possibilities. Firstly, the food matrix of soy flour may alter the bioavailability of daidzein. Dietary factors such as fiber and carbohydrate have been associated with differences in the metabolism of daidzein to equol [18-20]. Urinary recovery of equol is higher following the ingestion of tempeh when compared with homogeneous soymilk and textured vegetable protein. This suggests that the combination of a food matrix might protect daidzein from degradation until it reaches the large intestine where it can be metabolized to equol by the microflora [20]. Secondly, in this study, we only determined the isoflavone contents in both soy preparations as the forms of β-glycoside conjugates (daidzin and genistin) as well as aglycones (daidzein and genistein). However, soy flour and soy extract capsules might have contained some malonyl glycoside and acetyl glycoside conjugates of isoflavones, which were not measured in this study. We postulate that the proportions of malonyl glycoside and acetyl glycoside conjugates of daidzein in soy extract capsules may be greater than those in soy flour, resulting in better bioavailability after these conjugates are converted and absorbed as daidzein from the gastrointestinal tract.

The mean T_max _of daidzein and genistein from both soy preparations in this study was shorter than the values of 8–11 h (after ingestion of β-glycoside conjugates) as reported in previous studies [21,22]. This difference might result from variation in age, race, uptake rates, hydrolysis of glycosides by gut bacteria or gut wall enzymes, further metabolism (for example glucuronides within the liver), etc. The second peak demonstrated in the plasma concentration-time curves of daidzein and genistein possibly resulted from enterohepatic recirculation of the glucuronide and sulfate conjugates of isoflavones excreted in bile [22]. The elimination t_1/2 _of daidzein and genistein in this study was comparable to the values of 6–8 h from other studies [20-22].

It has been suggested that a daily intake of soy isoflavone extract containing 50/50 mg of genistin and daidzin [23] or 76 mg of isoflavones [24] can significantly decrease hot flushes in the group treated with soy products over the placebo. A meta-analysis of 38 clinical trials, which examined the relationship between soy protein intake and serum lipids, have shown that the consumption of soy in men and women is associated with a significant decrease in serum cholesterol, LDL and triglyceride levels [25]. In a randomized, double-blind, placebo-controlled trial, which examined the effects of dietary soy supplements containing 118 mg of isoflavones on the lipid profiles of men and postmenopausal women with relatively normal cholesterol levels, the LDL/HDL ratio decreases in the isoflavone treatment groups without any change in total cholesterol [26]. In addition, it has been found that those postmenopausal women with greatest phytoestrogen consumption have the highest bone mineral density (BMD) at the hip and spine. Subjects with the highest intake of isoflavones also have significantly lower levels of serum PTH, osteocalcin and urinary N-telopeptide [27]. Besides, isoflavone supplementation (61.8 mg of isoflavones) for four weeks shows potentially beneficial effects on bone metabolism and serum lipids in perimenopausal women in a randomized controlled trial [28]. Another trial has demonstrated that continuous dietary intake of isoflavones (37.3 mg/day) for ten weeks may inhibit postmenopausal osteoporosis [29]. Therefore, in our opinion, the total dose of isoflavones that benefits menopausal health is up to approximately 100 mg/day. Since our study revealed that the bioavailability of genistein from soy beverage and soy extract capsules was similar, and the bioavailability of daidzein was slightly (although statistically significant) lower than that of soy extract capsules, the amount of soy beverage, which provides the isoflavone bioavailability equivalent to soy isoflavone capsules containing 50/50 mg of daidzein and genistein, should be equal to approximately 5 cups/day (15 g of soy flour/cup). If one consumes other soy food or prepares a soy beverage in a higher concentration, the daily volume of consumption would be reduced. This inexpensive soy beverage is an appropriate alternative food supplementation compared to the more expensive soy extract capsules and HRT for postmenopausal Thai women, and is in line with Thailand's present socioeconomic status. However, the different proportion of daidzein and genistein in various soy preparations might affect beneficial outcomes for postmenopausal women. In this aspect, clinical studies should be investigated further.

## Conclusion

The bioavailability of daidzein, which was adjusted for the administered dose (AUC/dose) following a single oral administration of soy beverage, was slightly (but significantly) less than that of soy extract capsules, whereas, that of genistein from both soy preparations was comparable. There was also no difference in other pharmacokinetic parameters of daidzein and genistein, including C_max _adjusted for dose, T_max _and t_1/2 _between both soy preparations.

## Competing interests

The author(s) declare that they have no competing interests.

## Authors' contributions

EA performed the quantification of isoflavones and statistical analysis. ST supervised data collection and analysis, and drafted the manuscript. NR supervised the quantification of isoflavones. SP initiated the research question and participated in the selection of patients eligible for the study. CS participated in the design of the study and drafted the manuscript. All authors read and approved the final manuscript.

## Pre-publication history

The pre-publication history for this paper can be accessed here:



## References

[B1] Schneider HPG (2003). Menopause: The state of the art in research and management.

[B2] Women's Health Initiative Steering Committee (2004). Effects of conjugated equine estrogen in postmenopausal women with hysterectomy: the Women's Health Initiative randomized controlled trial. JAMA.

[B3] Warren MP (2004). A comparative review of the risks and benefits of hormone replacement therapy regimens. Am J Obstet Gynecol.

[B4] Warren MP, Shortle B, Dominguez JE (2002). Use of alternative therapies in menopause. Best Pract Res Clin Obstet Gynecol.

[B5] Song T, Barua K, Buseman G, Murphy PA (1998). Soy isoflavone analysis: quality control and a new internal standard. Am J Clin Nutr.

[B6] Setchell KD (2001). Soy isoflavones – benefits and risks from nature's selective estrogen receptor modulators (SERMs). J Am Coll Nutr.

[B7] Kuiper GG, Lemmen JG, Carlsson B, Corton JC, Safe SH, van der Saag PT, van der Burg B, Gustafsson JA (1998). Interaction of estrogenic chemicals and phytoestrogens with estrogen receptor beta. Endocrinology.

[B8] Kris-Etherton PM, Hecker KD, Bonanome A, Coval SM, Binkoski AE, Hilpert KF, Griel AE, Etherton TD (2002). Bioactive compounds in foods: their role in the prevention of cardiovascular disease and cancer. Am J Med.

[B9] Goldwyn S, Lazinsky A, Wei H (2000). Promotion of health by soy isoflavones: efficacy, benefit and safety concerns. Drug Metabol Drug Interact.

[B10] Wiseman H (2000). The therapeutic potential of phytoestrogens. Expert Opin Investig Drugs.

[B11] Adlercreutz H (1998). Epidemiology of phytoestrogens. Baillieres Clin Endocrinol Metab.

[B12] Nakamura Y, Tsuji S, Tonogai Y (2000). Determination of the levels of isoflavonoids in soybeans and soy-derived foods and estimation of isoflavonoids in the Japanese daily intake. J AOAC Int.

[B13] Thomas BF, Zeisel SH, Busby MG, Hill JM, Mitchell RA, Scheffler NM, Brown SS, Bloeden LT, Dix KJ, Jeffcoat AR (2001). Quantitative analysis of the principle soy isoflavones genistein, daidzein and glycitein, and their primary conjugated metabolites in human plasma and urine using reversed-phase high-performance liquid chromatography with ultraviolet detection. J Chromatogr B Biomed Sci.

[B14] Wang H, Murphy PA (1994). Isoflavone content in commercial soybean foods. J Agric Food Chem.

[B15] Barnes S, Kirk M, Coward L (1994). Isoflavones and their conjugates in soy foods: extraction conditions and analysis by HPLC-mass spectrometry. J Agric Food Chem.

[B16] Wang H, Murphy PA (1994). Isoflavone composition of American and Japanese soybeans in Iowa: effects of variety, crop year, and location. J Agric Food Chem.

[B17] Xu X, Wang H, Murphy PA, Hendrich S (2000). Neither background diet nor type of soy food affects short-term isoflavone bioavailability in women. J Nutr.

[B18] Tew BY, Xu X, Wang H, Murphy PA, Hendrich S (1996). A diet high in wheat fiber suppresses the bioavailability of isoflavones in a single meal fed to women. J Nutr.

[B19] Rowland I, Wiseman H, Sander T, Adlercreutz H, Bowey E (1999). Metabolism of estrogens and phytoestrogens: role of the gut microflora. Biochem Soc Trans.

[B20] Faughnan MS, Hawdon A, Ah-Singh E, Brown J, Millward DJ, Cassidy A (2004). Urinary isoflavone kinetics: the effect of age, gender, food matrix and chemical composition. Br J Nutr.

[B21] Setchell KD, Brown NM, Desai P, Zimmer-Nechemias L, Wolfe BE, Brashear WT, Kirschner AS, Cassidy A, Heubi JE (2001). Bioavailability of pure isoflavones in healthy humans and analysis of commercial soy isoflavone supplements. J Nutr.

[B22] Watanabe S, Yamaguchi M, Sobue T, Takahashi T, Miura T, Arai Y, Mazur W, Wahala K, Adlercreutz H (1998). Pharmacokinetics of soybean isoflavones in plasma, urine and feces of men after ingestion of 60 g baked soynean powder (kinako). J Nutr.

[B23] Upmalis D, Lobo R, Bradley L, Warren M, Cone FL, Lamia CA (2000). Vasomotor symptom relief by soy isoflavone extract tablets on postmenopausal women: a multicenter, double-blind, randomized, placebo-controlled study. Menopause.

[B24] Albertazzi P, Pansini F, Bonaccorsi G, Alakel L (1998). The effect of dietary soy supplementation on hot flushes. Obstet Gynecol.

[B25] Anderson J, Johnston B, Cook-Newell M (1995). Meta-analysis of the effects of soy protein intake on serum lipids. N Engl J Med.

[B26] Teede H, Dalais F, Kotsopoulos D, Liang Y, Davis S, McGrath B (2001). Dietaty soy has both beneficial and potentially adverse cardiovascular effects: a placebo-controlled study in men and postmenopausal women. J Clin Endocrinol Metab.

[B27] Lauderdale DS, Jacobsen SJ, Furner SE, Levy PS, Brody JA, Goldberg J (1997). Hip fracture incidence among elderly Asian-American populations. Am J Epidemiol.

[B28] Uesugi T, Fukui Y, Yamori Y (2002). Beneficial effects of soybean isoflavone supplementation on bone metabolism and serum lipids in postmenopausal Japanese women: a four-week study. J Am Coll Nutr.

[B29] Yamori Y, Moriguchi EH, Teramoto T, Miura A, Fukui Y, Honda KI, Fukui M, Nara Y, Taira K, Moriguchi Y (2002). Soybean isoflavones reduce postmenopausal bone resorption in female Japanese immigrants in Brazil: a ten-week study. J Am Coll Nutr.

